# Quantitative myocardial perfusion response to adenosine and regadenoson in patients with suspected coronary artery disease

**DOI:** 10.1007/s12350-021-02731-6

**Published:** 2021-08-12

**Authors:** Tanja Kero, Antti Saraste, Bo Lagerqvist, Jens Sörensen, Essi Pikkarainen, Mark Lubberink, Juhani Knuuti

**Affiliations:** 1 Medical Imaging Centre, Uppsala University Hospital, Uppsala, Sweden; 2 Department of Cardiology, Uppsala University Hospital, Uppsala, Sweden; 3 Medical Physics, Uppsala University Hospital, Uppsala, Sweden; 4 Department of Surgical Sciences/Radiology, Uppsala University, Uppsala, Sweden; 5 Department of Medical Sciences, Uppsala University, Uppsala, Sweden; 6 Turku PET Centre, Turku, Finland; 7 Heart Centre, Turku University Hospital and University of Turku, Turku, Finland

**Keywords:** Modalities, Myocardial blood flow, Vasodilators

## Abstract

**Background:**

The aim of the present study was to compare the quantitative flow responses of regadenoson against adenosine using cardiac ^15^O-water PET imaging in patients with suspected or known coronary artery disease (CAD).

**Methods:**

Hyperemic myocardial blood flow (MBF) after adenosine and regadenoson was compared using correlation and Bland–Altman analysis in 21 patients who underwent rest and adenosine ^15^O-water PET scans followed by rest and regadenoson ^15^O-water PET scans.

**Results:**

Global mean (± SD) MBF values at rest and stress were 0.92 ± 0.27 and 2.68 ± 0.80 mL·g·min for the adenosine study and 0.95 ± 0.29 and 2.76 ± 0.79 mL·g·min for the regadenoson study (*P* = 0.55 and *P =* 0.49). The correlations between global and regional adenosine- and regadenoson-based stress MBF were strong (*r* = 0.80 and *r* = 0.77). The biases were small for both global and regional MBF comparisons (0.08 and 0.09 mL·min·g), but the limits of agreement were wide for stress MBF.

**Conclusion:**

The correlation between regadenoson- and adenosine-induced hyperemic MBF was strong but the agreement was only moderate indicating that established cut-off values for ^15^0-water PET should be used cautiously if using regadenoson as vasodilator.

**Supplementary Information:**

The online version contains supplementary material available at 10.1007/s12350-021-02731-6.

## Chinese Abstract

**背景:**

本文目的是对疑似或确诊的冠状动脉心脏病患者行15O水 PET心脏显像, 比较瑞加德松和腺苷影响心肌血流反应的定量研究。

**方法:**

21例患者依次行静息-腺苷15O水 PET心脏显像和静息-瑞加德松15O水 PET心脏显像, 对应用腺苷和瑞加德松后的两次充血性心肌血流量(MBF)行相关性和Bland Altman分析。

**结果:**

腺苷组静息和负荷下的整体MBF分别为0.92±0.27和2.68±0.80 mL/g/min, 瑞加德松组静息和负荷下的整体MBF分别为0.95±0.29和2.76±0.79mL/g/min (p=0.55 和 p=0.49)。腺苷和瑞加德松负荷状态下的整体和局部MBF之间的相关性很强(r=0.80 和 r=0.77)。静息状态下的整体和局部MBF两者偏倚均较小(0.08 and 0.09mL/min/g), 但对于负荷下的整体和局部MBF, 两组一致性限度值较宽。

**结论:**

使用瑞加德松和腺苷后的充血性MBF之间的相关性很强, 但一致性一般。该研究结果表明在应用瑞加德松行15O水 PET心脏显像时, 应该谨慎使用之前建立的临界值。

## Introduction

Historically, the most common pharmacological stressors for myocardial perfusion imaging (MPI) are dipyridamole and adenosine, which cause coronary vasodilatation by stimulating the A_2A_ receptor. Due to the stimulation of A_1_, A_2B_, and A_3_ receptors, adenosine is also associated with several side effects like general discomfort, chest pain, hypotension, bronchospasm, or atrioventricular block.^1^ Regadenoson is a newer, selective A_2A_ receptor agonist causing coronary vasodilatation, which avoids activation of other adenosine receptors that cause side effects, resulting in a better tolerability than for adenosine. Regadenoson is easy to use; it is injected intravenously as a bolus with the same dose to all patients; thus, there is no need for weight determination and dose calculation, reducing the risk of dosing errors.^2^ Several clinical studies have demonstrated the efficacy and safety of regadenoson and it is increasingly being used for MPI with SPECT.^2^–^5^ Positron emission tomography (PET) enables quantitative measurements of myocardial blood flow (MBF) and myocardial flow reserve (MFR), and with recent developments and improved availability of PET technology, there has been a growing interest in translation of quantitative flow analysis from a research setting to routine clinical practice. A few PET studies have measured the absolute myocardial flow responses to regadenoson using ^82^Rb and the results have been conflicting; while the values have been comparable to dipyridamole at group level,^6^,^7^ a more recent direct comparison, however, demonstrated that compared to dipyridamole, regadenoson administered to the same patients achieved only 80% of maximal hyperemia.^8^ Furthermore, ^82^Rb is not an ideal perfusion tracer because of its limited extraction especially at high blood flow values. For accurate MBF measurements by PET, an ideal PET tracer should have a high first-pass extraction fraction at high blood flow.^9^^15^O-water meets this criterion and is considered to be the gold standard for non-invasive quantitative measurements of MBF.^10^–^12^ The flow responses to adenosine have been investigated in numerous studies and recently also normal ranges and optimal cut-off limits have been defined for ^15^O-water PET in diagnostic work in patients with suspected CAD.^13^ When quantification is applied in clinical work, it is critical to know whether the same cut-off values could be used also with regadenoson but there are no studies comparing absolute flow response to regadenoson against adenosine with ^15^O-water PET in the same patients. The aim of the present study was therefore to compare the quantitative flow responses of regadenoson against adenosine using cardiac ^15^O-water PET imaging in a paired design in patients with suspected or known CAD.

## Materials and Methods

### Subjects

26 patients were included in this prospective, two-center study: 9 patients in Turku and 17 patients in Uppsala. The patients had suspected or known CAD and were referred for a coronary angiography or an ^15^O-water PET-CT study for evaluation of MBF based on clinical indications. Patients with prior ST-elevation myocardial infarction (STEMI), prior coronary artery bypass grafting (CABG), left ventricular ejection fraction < 40%, cardiomyopathy, or severe valvular or pulmonary disease were excluded as were patients with any contraindications for adenosine or regadenoson.

Written informed consent was obtained from all subjects and the study was performed with permission from the regional boards of medical ethics in Turku and in Uppsala.

### Simulation Study

Simulations were done to assess the effect of timing of ^15^O-water administration after regadenoson injection on the accuracy of estimated MBF and to assess the effect of variations on time to and duration of the hyperemic plateau. The range of simulated variations was based on previously published studies.^14^,^15^

First, MBF response to regadenoson was modeled by a linear increase from 1 to 3 mL·g·min during 30 seconds, followed by a 3 minutes plateau and an exponential reduction with a 5 minutes half-life. Myocardial tissue time-activity curves (TACs) were numerically simulated using arterial and right-ventricular curves from a typical patient, applying a perfusable tissue fraction (PTF) of 0.7 g·mL, left- and right-ventricular spill-over fractions of 0.1, and scan start between 0 and 90 seconds after regadenoson administration in increments of 1 seconds (see Equation 1 below). These TACs were fitted to the operational equation of the single-tissue compartment model (see below) using non-linear regression, and bias in fitted MBF relative to simulated MBF versus time interval between regadenoson and ^15^O-water injections was calculated.

Then, simulations were repeated with violations of the underlying assumptions of a 30 seconds build-up phase and 3 minutes plateau phase. MBF response to regadenoson was modeled assuming a linear increase in MBF from 1 to 3 mL·g·min during a build-up phase lasting 0-90 seconds and plateau durations between 0 and 3 minutes in increments of 10 seconds, with a scan start at 30 seconds. Myocardial TACs were numerically simulated as above and fitted to the single-tissue compartment model, and bias in fitted MBF relative to the true hyperemic MBF of 3 mL·g·min as a function of plateau duration and build-up phase duration was calculated. Simulated scan duration was 4 minutes, and no noise was added to the simulated TACs as we wanted to isolate the effect of time to and duration of the hyperemic plateau.

### Scan Procedure

All subjects underwent rest and adenosine stress ^15^O-water PET scans according to clinical routine at each site, followed by rest and regadenoson stress ^15^O-water PET scans. The subjects were instructed to abstain from caffeine for 24 hours before imaging. Coronary computed tomography (CT) angiography or invasive coronary angiography was carried out according to the standard clinical procedures; in Turku, CT angiography was carried out at the same occasion as the PET study preceding the PET scan. In Uppsala, invasive coronary angiography was carried out on a separate day after the PET scan.

In Turku, PET scans were either performed on a VCT PET/CT (GE Healthcare, Waukesha) in 2D acquisition mode or a Discovery 690 (GE Healthcare) in 3D acquisition mode. ^15^O-water (900 to 1100 MBq in 2D or 400-500 MBq in 3D) was automatically injected using a radiowater generator (Hidex Oy, Finland) as an intravenous bolus over 15 seconds. A 4-min and 40-sec dynamic acquisition (14 × 5 seconds, 3 × 10 seconds, 3 × 20 seconds, and 4 × 30 seconds) of the heart was performed at rest and stress. In Uppsala, 400 MBq ^15^O-water was injected at rest and at stress with a Medrad contrast injector followed by 35 mL saline (10 mL at 0.8 mL·s + 30 mL at 2 mL·s). A 6-min 3D dynamic acquisition (1 × 10, 8 × 5, 4 × 10, 2 × 15, 3 × 20, 2 × 30, 2 × 60 seconds) was performed using a GE Discovery ST PET/CT scanner (GE Healthcare, Waukesha).

At both sites, a single low-dose CT scan was acquired before the resting PET scan in order to correct for photon attenuation.

For the adenosine study, adenosine infusion was started 2 minutes before the start of the PET scan and infused at 140 µg ·kg body weight per minute until the end of the acquisition. In the regadenoson study, Rapiscan (400 µg regadenoson/5 ml) was injected intravenously during 10 seconds followed by a 10-second saline flush (5 ml). ^15^O-water was injected 10–20 seconds after the saline flush and followed by dynamic acquisition according to standard ^15^O-water protocol.

### Image Reconstruction and Data Analysis

The PET images were reconstructed using standard reconstruction parameters of the scanners (Uppsala: ordered subset expectation maximization (OSEM) with 2 iterations, 21 subsets, and a 5 mm Gaussian post-filter; Turku: OSEM with 2 iterations, 20 subsets, and a 6 mm Gaussian postfilter), applying all necessary corrections including CT-based attenuation correction. The PET data were analyzed semi-automatically with aQuant software (MedTrace Pharma A/S, Lyngby, Denmark); myocardial segment VOIs were drawn over the left ventricle based on the 17-segment model of the American Heart Association^16^ generating MBF values for the entire left ventricle, in all 17 myocardial segments and in three regions corresponding to the three main coronary artery territories. The calculation of MBF at regional and global level was based on non-linear regression of the solution of a single-tissue compartment model to the mean time-activity curve, using an arterial input function from cluster analysis comprising left atrial and ventricular cavities and ascending aorta and with correction for spillover from left and right ventricular cavities into the myocardium:

1 $$C_{{{\text{PET}}}} \left( t \right) = {\text{PTF}} \cdot {\text{MBF}} \cdot C_{A} \left( t \right) \otimes e^{{\frac{{ - {\text{MBF}}}}{{V_{T} }}t}}  + V_{{{\text{LV}}}} C_{A} \left( t \right) + V_{{{\text{RV}}}} C_{{{\text{RV}}}} (t). $$

Here, *C*_PET_(*t*) is the radioactivity concentration as measured in a voxel or region by PET, PTF is the perfusable tissue fraction, and *V*_T_ is the distribution volume of water, fixed to 0.91 mL·g. *C*_A_(*t*) and *C*_RV_(*t*) are the radioactivity concentrations in arterial blood and in the right ventricular cavity, respectively, and *V*_LV_ and *V*_RV_ are the left- and right-ventricular spillover fractions.^17^ The software uses only the first 4 minutes of the acquired PET data.

The PET results were rated according to the previously suggested method by Danad et al.^13^: PET was rated as abnormal at patient or regional level if there were at least two adjacent segments with stress MBF below the cut-off value of 2.30 mL·g·min in the entire myocardial wall or in the specific coronary artery region, respectively.

The coronary angiographies were visually inspected and a stenosis ≥ 70% was considered significant.

### Statistical Analyses

Statistical analyses were performed using the IBM SPSS Statistics (version 26.0 for Macintosh, Armonk, NY: IBM corp, USA) and GraphPad Prism (version 8.3.0 Macintosh Version, GraphPad Software, San Diego, California, USA).

Continuous variables are presented as mean values ± standard deviation (SD), except were stated. Data were tested for normality with Shapiro–Wilk test and comparison of means was performed by paired *T* test. Correlation and agreement between adenosine- and regadenoson-based global and regional (the three coronary artery regions) MBF and MFR values were assessed using Deming regression (assuming equal uncertainties for *x* and *y*), Pearson correlation coefficient, and Bland–Altman analysis. A two-sided *P*-value of less than 0.05 was considered significant. Cohen’s kappa statistic was used to measure agreement between categorical variables. Sensitivity and specificity to detect obstructive CAD (defined as ≥ 70% stenosis on coronary angiography) were calculated for both adenosine and regadenoson MBF.

Reproducibility coefficient (RDC) defined as the least significant difference between two repeated measurements taken under different conditions^18^ was calculated for MBF as 1.96 times the SD of a difference between two measurements’ MBF in the adenosine study and MBF in the regadenoson study.

## Results

### Study Population

26 patients with known (N = 7) or suspected CAD were included in the study: 17 patients in Uppsala and 9 patients in Turku. All stress scans were completed successfully and none of the patients had significant adverse effects requiring intervention, such as AV block or bronchospasm. Data from three patients were excluded; one patient could not complete the PET studies due to back pain and in two patients, the PET data could not be analyzed due to technical error during PET scan or due to extensive artifacts. Table 1 shows baseline characteristics of the 23 patients who completed the study. 15 of the subjects underwent rest and adenosine stress ^15^O-water PET scans followed by rest and regadenoson stress ^15^O-water PET scans the same day and 8 subjects underwent the two PET studies within 18 days (range 4-18 days). Two of the patients showed no hyperemic response to one of the vasodilators: one patient had no hyperemic effect of adenosine (MFR = 1.0) but with regadenoson, MFR was 1.9. In this patient, regadenoson was administered approximately 60 minutes after the adenosine study, and it is probable that the differences in hyperemic response between the two studies were due to caffeine effects. Another of our patients had a normal flow increase with adenosine (MFR = 3.6) but no flow increase with regadenoson (MFR = 1.0). In this case, the regadenoson study was performed 18 days after the adenosine study, and it is probable that the patient refrained from caffeine before the first PET study but not the other. These two patients were excluded from further analysis.

**Table 1:** Hemodynamic and global MBF values

Age (years)	66 (range 38-79)
Male	18 (78%)
Body mass index (kg·m^2^)	26 (range 20-31)
Current smoking	2 (9%)
Previous smoking	13 (57%)
Previous PCI	6 (26%)
Previous NSTEMI	1 (4%)
Diabetes	4 (17%)
Hypertension	17 (74%)
Hyperlipidemia	17 (74%)
Betablockers	10 (43%)
Statins	14 (61%)
ACE-inhibitors/ARB	11 (48%)
Calcium channel blockers	7 (30%)
Long-acting nitrates	5 (22%)
Acetylsalicylic acid	16 (70%)

*PCI* percutaneous coronary intervention, *NSTEMI* non-ST-elevation myocardial infarction, *ACE* angiotensin-converting enzyme, *ARB* angiotensin receptor blocker

For the remaining 21 patients, systolic blood pressure, heart rate at rest, and rate pressure product (RPP) were comparable between the adenosine and the regadenoson studies, whereas the heart rate was slightly higher during regadenoson stress than during adenosine stress as shown in Table 2.

**Table 2:** Hemodynamic and global MBF values

	Adenosine rest	Regadenoson rest	*P*	Adenosine stress	Regadenoson stress	*P*
SBP	123 ± 21	121 ± 17	0.61	127 ± 18	126 ± 15	0.81
HR	65 ± 15	65 ± 14	0.89	86 ± 13	91 ± 16	0.02
RPP	7993 ± 2167	7928 ± 2264	0.88	10896 ± 2252	11509 ± 2948	0.16
Global MBF	0.92 ± 0.27	0.95 ± 0.29	0.55	2.68 ± 0.80	2.76 ± 0.79	0.49
Global MBFcorr	1.16 ± 0.20	1.26 ± 0.40	0.15			

*SBP* systolic blood pressure (mmHg), *HR* heart rate (beats per minute), *RPP* rate pressure product (SBP × HR), *MBF* myocardial blood flow (mL·cm^3^·min), *MBFcorr* myocardial blood flow corrected for RPP = (MBF/RPP) x 10^4^

### Simulation Study

**Figure 1:**
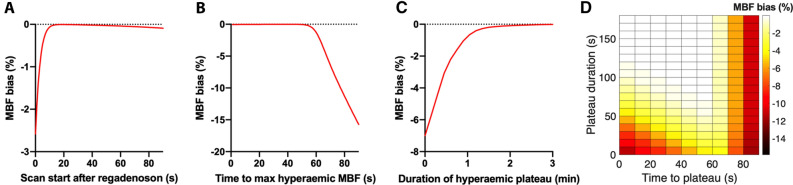
**a** Bias in MBF versus time difference between regadenoson injection and ^15^O-water administration assuming a 30 second linear build-up of MBF followed by a 3 minutes hyperemic plateau phase. **b** Bias in MBF versus duration of the build-up phase, assuming ^15^O-water administration 30 seconds after regadenoson injection and a 3 minutes hyperemic plateau. **c** Bias in MBF versus duration of hyperemic plateau phase assuming a 30 seconds linear build-up of MBF and ^15^O-water administration 30 seconds after regadenoson injection. **d** Effects of both duration of build-up phase and plateau phase on bias in MBF assuming 30 seconds after regadenoson injection

Figure 1 shows the results of the simulations done to assess the effect of timing of ^15^O-water administration after regadenoson injection on the accuracy of estimated MBF and to assess the effect of variations on time to and duration of the hyperemic plateau. Assuming a 30 seconds build-up phase to hyperemic MBF and a 3 minutes plateau, a scan start later than 15 seconds after regadenoson injection would lead to negligible bias (Figure 1a). Figure 1b–d shows that inter-individual variations in build-up and plateau phase durations result in negligible MBF bias when starting ^15^O-water administration at 30 seconds after regadenoson injection, as long as build-up phase duration is less than 60 seconds and plateau phase duration is more than 60 seconds. However, longer build-up phase durations and/or shorter plateau phase durations result in a rapidly increasing negative bias in MBF values.

### Myocardial Perfusion

**Figure 2:**
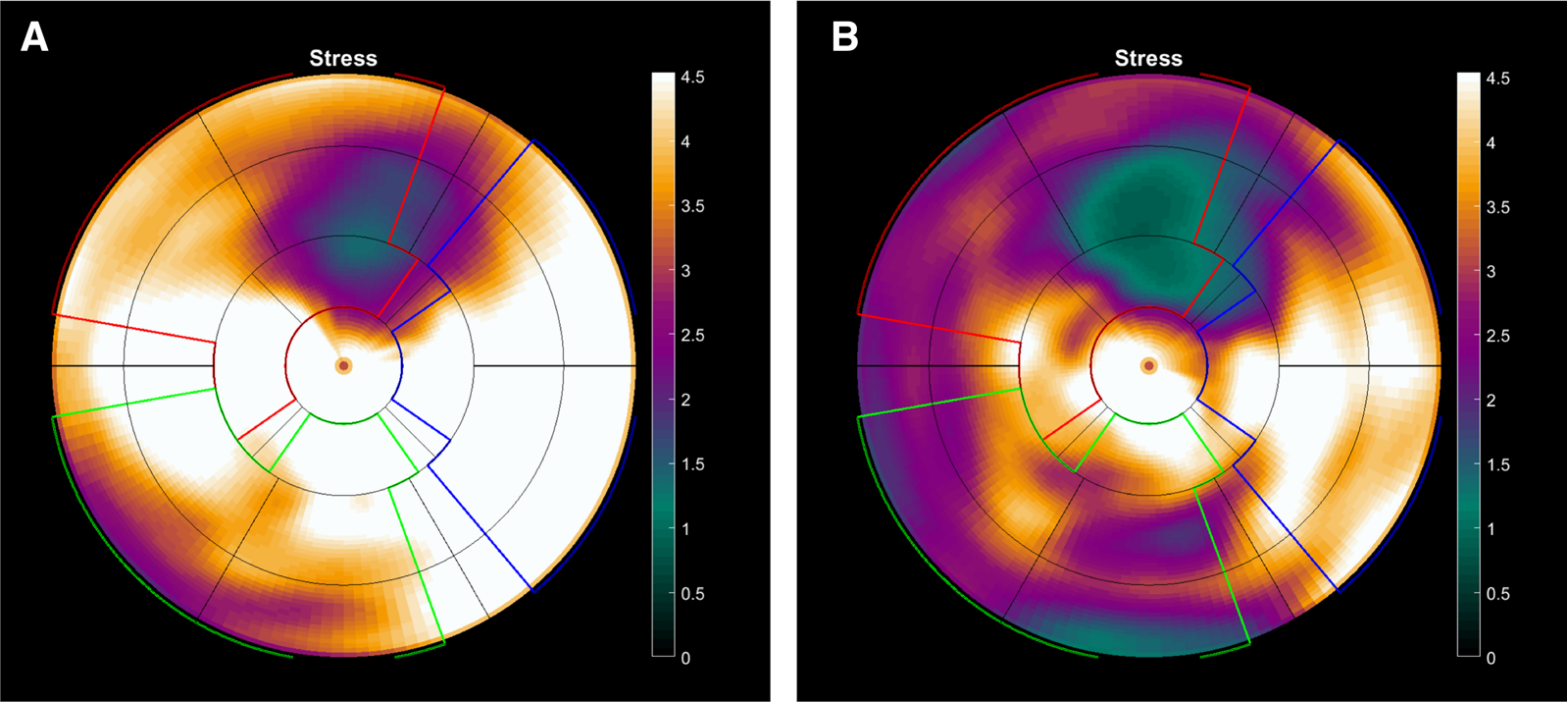
Polarplots of stress MBF from adenosine (s) and regadenoson (b) PET MPI from a 70 years old male with hypertension and history of smoking, on current medication with acetylsalicylic acid and betablockers. ^15^O-water PET was requested because of suspected CAD due to typical angina. Adenosine and regadenoson ^15^O-water PET were performed the same day. Adenosine PET MPI (**a**) showed a stress perfusion defect in the anterior myocardial wall with perfusion defect size approximately two segments with average stress MBF in the perfusion defect 2.07 mL·min·g. The average stress MBF was 3.20 mL·min·g in the LAD region. Global stress MBF was 3.82 mL·min·g. Regadenoson PET MPI (**b**) showed a stress perfusion defect in the anterior myocardial wall with perfusion defect size approximately three segments with average stress MBF in the perfusion defect 1.63 mL·min·g. The average stress MBF was 2.07 mL·min·g in the LAD region. Global stress MBF was 2.55 mL·min·g. In the regadenoson PET MPI (**b**), a small perfusion defect could also be seen in the inferior wall (two basal inferoseptal segments with average stress MBF 2.02 L·min·g), whereas the average stress MBF was 2.75 mL·min·g in these two segments in the adenosine PET (**a**). Invasive coronary angiography showed a 50%-70% stenosis in the LAD coronary artery and a 70%-90% stenosis in the RCA

Figure 2 shows images from adenosine and regadenoson ^15^O-water PET in one patient where both stressors showed a perfusion defect in the LAD region (albeit smaller defect size with adenosine) but discordant findings in the RCA region. In this patient, the stress MBF values were overall slightly higher with adenosine than with regadenoson.

**Figure 3:**
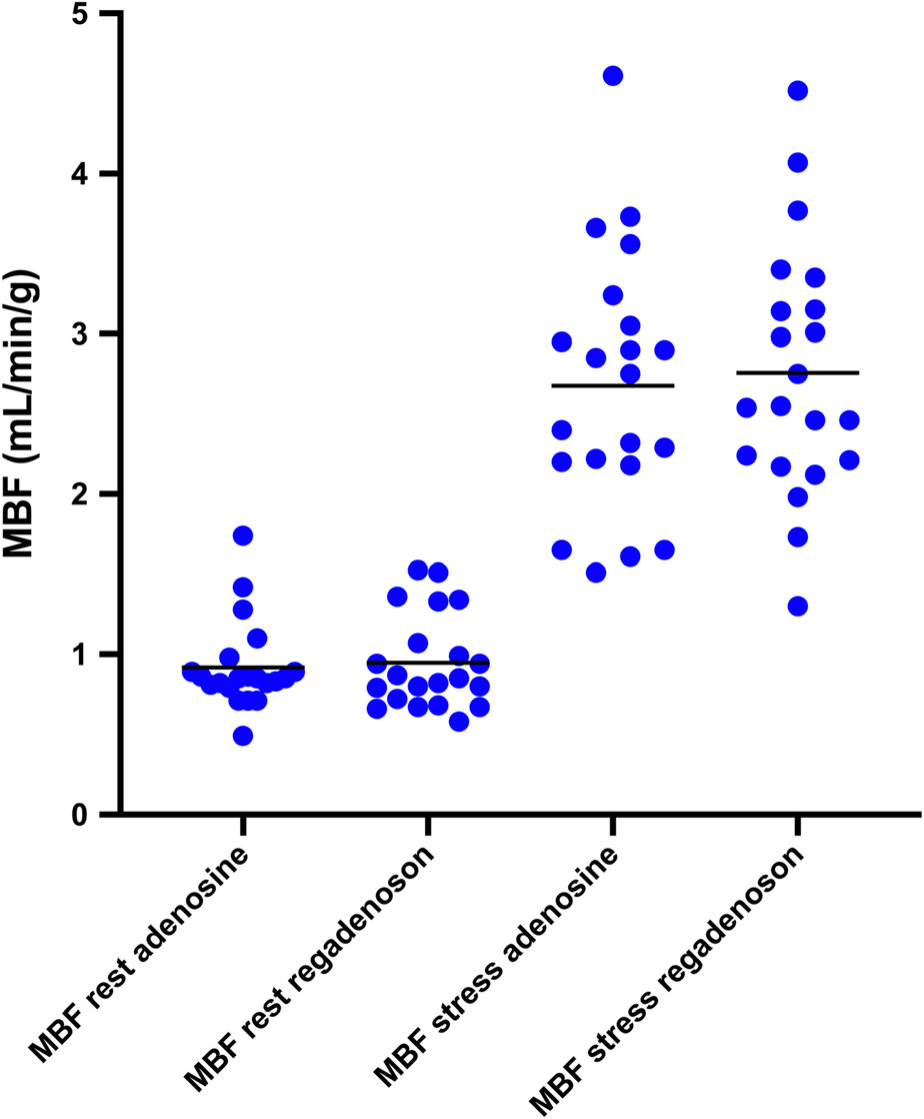
Scatter plot of global rest and stress MBF from adenosine and regadenoson PET. The horizontal lines show the mean values

**Figure 4:**
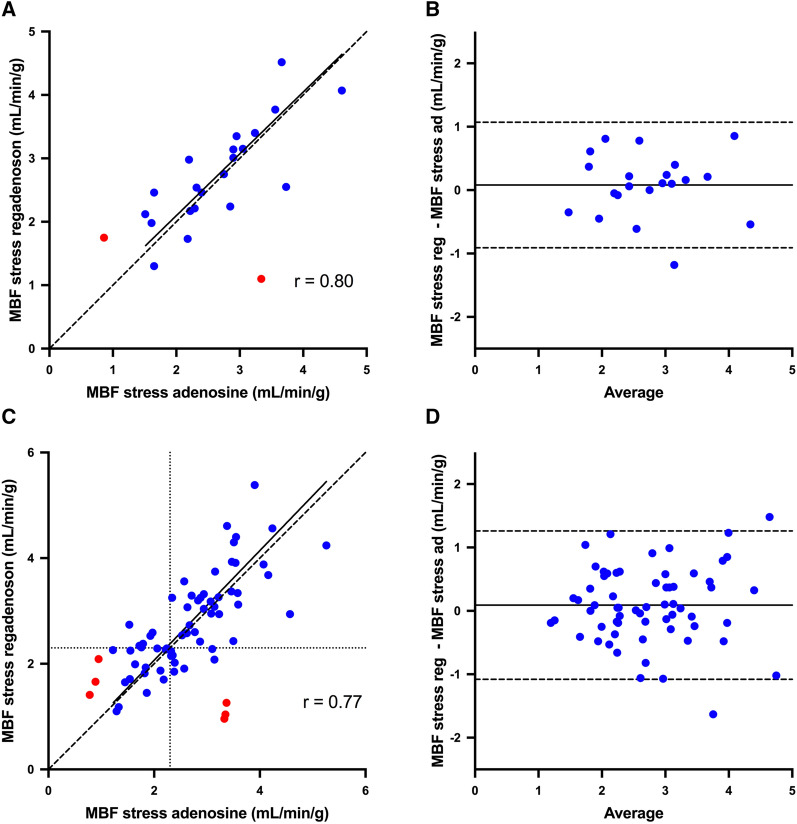
Correlation (**a**, **c**) and Bland–Altman plots (**b**, **d**) of global MBF at adenosine stress versus regadenoson stress (**a**, **b**) and regional MBF at adenosine stress vs regadenoson stress (**c**, **d**). The red dots are the MBF values of the two subjects without hyperemic response to one of the vasodilators that were excluded from further analysis. The solid lines in a and c are Deming regression slopes and the dashed lines are lines of identity. The dotted lines in c indicate MBF=2.3 mL·min·g (indicating the optimal cut-off for ^15^O-water PET stress MBF according to Danad et al.^13^). The solid lines in b and d indicate the mean difference (bias), whereas the dashed lines show the limits of agreement. Regression slopes are 0.97 (0.51-1.44) and 1.04 (0.75-1.33) in a and c. Bias (limits of agreement) are 0.08 (− 0.91 to 1.07) and 0.09 (− 1.08 to 1.26) in b and d. Biases were not statistically significant for global MBF (*P =* 0.49) or regional MBF (*P =* 0.23)

For the 21 study patients, global mean (± SD) MBF values at rest and stress were 0.92 ± 0.27 and 2.68 ± 0.80 mL·g·min for the adenosine study and 0.95 ± 0.29 and 2.76 ± 0.79 mL·g·min for the regadenoson study (*P =* 0.55 and *P =* 0.49) (Figure 3). The relations between global and regional stress MBF from the adenosine and regadenoson studies are shown in Figure 4.

The Pearson correlation coefficients between RPP and global MBF at rest were 0.74 and 0.30 and at stress 0.36 and 0.57 for adenosine and regadenoson, respectively.

**Figure 5:**
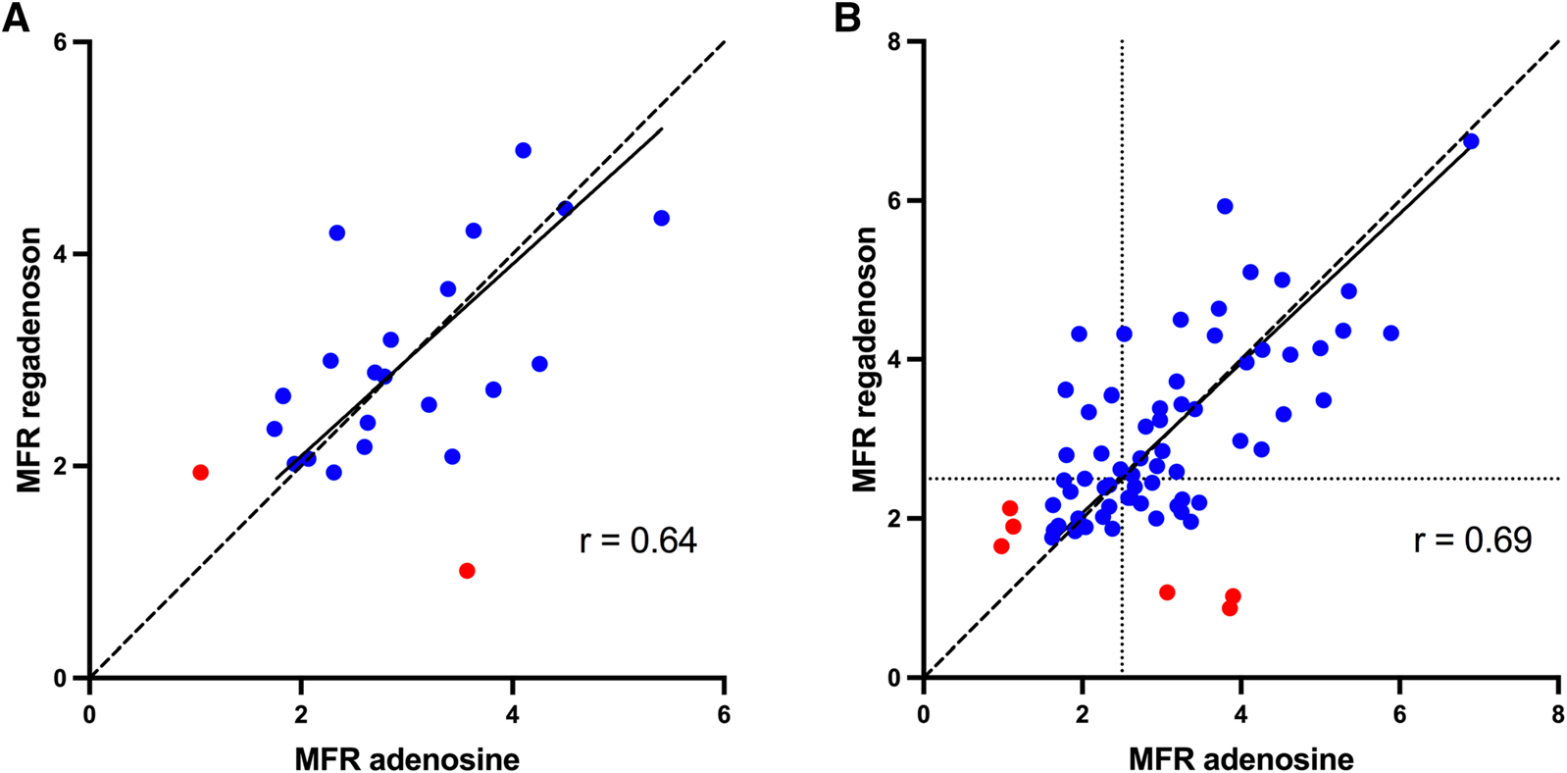
Correlation of global and regional MFR from adenosine PET versus regadenoson PET (**a** and **b**). The solid lines are Deming regression slopes and the dashed lines are lines of identity. The red dots are the MFR values of the two subjects without hyperemic response to one of the vasodilators that were excluded from further analysis. The dotted lines in b indicate MFR=2.5 (indicating the optimal cut-off for ^15^O-water PET MFR according to Danad et al.^13^). Regression slopes are 0.91 (0.32-1.49) and 0.94 (0.70-1.18) in **a** and **b**

Global mean (± SD) MFR values were 3.04 ± 0.98 for the adenosine study and 3.03 ± 0.92 for the regadenoson study (*P =* 0.97). The relations between global and regional MFR from the adenosine and regadenoson studies are shown in Figure 5.

Using the previously established cut-off value of 2.3 mL·min·g for stress MBF^13^, on a patient level, 18 out of 21 patients had concordant PET diagnosis results (86%; Kappa coefficient = 0.67) and 3 patients had discordant results; 5 patients had normal PET MBF results with both adenosine and regadenoson and 13 patients had abnormal results (defined as at least one perfusion defect with minimum size of two segments with stress MBF < 2.30 mL·min·g) with both vasodilators; one patient had normal adenosine MBF but abnormal regadenoson MBF and two patients had abnormal adenosine MBF results but normal regadenoson MBF.

On a regional level, 50 out of 63 coronary regions had concordant results (79%; Kappa coefficient = 0.57) and 13 had discordant results; 32 regions had normal stress MBF with both vasodilators, 18 regions had abnormal stress MBF with both vasodilators, 6 regions had normal stress MBF with adenosine but abnormal with regadenoson, and 7 regions had abnormal stress MBF with adenosine but normal with regadenoson.

### Coronary Angiography

In Turku, CT angiography was performed in 7 patients (the same day as adenosine PET) and one of these patients also underwent ICA (82 days after adenosine PET and CTA). In Uppsala, invasive coronary angiography was performed in 10 patients within 6-80 days after the adenosine PET.

Seven patients (41%) were found to have obstructive CAD with a significant stenosis in at least one of the coronary arteries. Both adenosine and regadenoson ^15^O-water PET correctly identified obstructive CAD in all of these patients (sensitivity 100%).

On a regional level, 10 of the coronary arteries (20%) had evidence of a significant stenosis. Adenosine ^15^O-water PET correctly identified 8 of the 10 arteries with significant stenosis (sensitivity 80%) and regadenoson correctly identified 9 of the 10 arteries with significant stenosis (sensitivity 90%). Specificities at patient level were 40% and 50% and at regional level 68% and 71% for adenosine and regadenoson PET MBF, respectively.

Coronary angiography findings in the patients with discordant PET MBF findings were on a per-patient level (where three patients were categorized discordantly); one patient who had normal adenosine MBF but abnormal regadenoson MBF had no evidence of obstructive CAD on coronary angiography and two patients who had abnormal adenosine MBF results but normal regadenoson MBF did not have any significant stenosis on angiography. On a regional level, 13 out of the total 63 coronary artery regions were categorized discordantly by PET MBF and all of these were also assessed with coronary angiography: 6 regions had normal stress MBF with adenosine but abnormal with regadenoson: angiography did not identify a significant stenosis in 4 of these regions (defined as ≥ 70% stenosis) but showed significant stenosis in 2 regions and 7 regions had abnormal stress MBF with adenosine but normal with regadenoson: angiography could not identify a significant stenosis in 6 of these regions but showed a significant stenosis in one region).

## Discussion

This paired study comparing the quantitative flow responses of regadenoson against adenosine using cardiac ^15^O-water PET imaging in patients with known or suspected CAD showed that regadenoson achieved comparable hyperemia to adenosine. The correlations between global and regional adenosine-based and regadenoson-based stress MBF values were strong (*r* = 0.80 and *r* = 0.77); the biases were very small for both global and regional MBF comparisons (0.08 and 0.09 mL·min·g), but the limits of agreement were fairly wide for both global (− 0.91 to 1.07) and regional (− 1.08 to 1.26) stress MBF, indicating a moderate overall agreement.

Regadenoson has been compared to dipyridamole or adenosine in several earlier studies with different modalities including SPECT,^3^,^5^ PET,^6^–^8^,^19^ magnetic resonance imaging (MRI),^20^,^21^ and coronary fractional flow reserve measurements (FFR)^22^–^25^. Regadenoson already has an established role in SPECT MPI with large studies showing that regadenoson provides diagnostic information comparable to a standard adenosine infusion.^3^,^26^ However, the SPECT studies were based on relative uptake images and have not quantified myocardial blood flow in absolute terms, and so the results from those studies cannot be compared to ours nor assumed to be valid for quantitative MBF. Quantitative or semi-quantitative CMR^20^,^21^ and FFR studies^22^–^25^ have shown similar flow response for regadenoson as adenosine. A few studies have measured the absolute myocardial flow responses to regadenoson using ^82^Rb PET, and these values have been comparable to dipyridamole at group level.^6^,^7^ Only one quantitative cardiac PET study using a paired design has been published; in which, using ^82^Rb PET, regadenoson achieved only 80% of maximal hyperemia as compared to dipyridamole.^8^ That study further showed that a more delayed radionuclide injection after the regadenoson bolus improved the hyperemic effect of regadenoson, suggesting that the timing between regadenoson and the tracer administration can affect the flow results.

Different modalities, flow tracers, and quantification models influence the quantitative MBF results; results from studies using one tracer or modality may not be directly applicable to another. Accurate MBF quantification requires a tracer with a high first-pass myocardial extraction fraction and absence of roll-off at high flow.^9^^82^Rb has the lowest extraction fraction among the PET perfusion tracers which is why it is far from ideal for accurate quantification of MBF and earlier results from comparisons of hyperemic MBF with ^82^Rb may not be directly applicable for other perfusion tracers with higher extraction fraction. ^15^O-water has no roll-off phenomenon at high flow and is considered the ideal PET tracer for quantification of MBF. ^13^NH_3_ is another excellent PET perfusion tracer with high extraction. Although there are a few cardiac PET studies in which regadenoson has been used together with ^15^O-water^27^ or ^13^NH_3_ PET,^28^–^30^ there are no previous comparisons of the hyperemic effect of regadenoson versus adenosine or dipyridamole using an ideal PET perfusion tracer.

For all PET tracers except ^15^O-water, MBF is determined based on the uptake rate of the tracer, representing transmural MBF (MBFt). With ^15^O-water, however, MBF is determined based on its efflux rate rather than its uptake rate. To test whether our results would have been different if determining MBF from the uptake rate, we also evaluated global stress MBFt (MBF*PTF), but found no significant difference in hyperemic response between adenosine or regadenoson using this parameter either (2.20 ± 0.75 vs 2.23 ± 0.61 mL·min·g, adenosine and regadenoson stress MBFt, respectively; *P =* 0.75. Mean difference (bias) of global MBFt was 0.04 and limits of agreement were − 0.99 to 1.07 mL·min·g and the reproducibility coefficient was 1.03 or 47% of mean).

Adenosine is a short-acting vasodilator and the hyperemic effect is ensured with a continuous infusion during the imaging. Regadenoson has a longer vasodilating effect and is administered as a bolus injection, which requires timing of the MBF quantification to the hyperemic plateau. Lieu at al. earlier showed that the increase in intracoronary blood flow velocity caused by a regadenoson bolus administration reached peak values within 0.5 minutes^14^ and that the mean duration of the increase in blood flow velocity of 2.5-fold or greater was 2.3-2.4 minutes. Van Nunen et al. compared the hyperemic effect of a regadenoson bolus injection to an adenosine infusion for inducing hyperemia in the measurement of fractional flow reserve (FFR).^25^ They found that the onset of maximum hyperemia was rapid (30±13 seconds after peripheral injection) but that the duration of the hyperemic plateau for regadenoson was highly variable among individuals with durations between 10 seconds and more than 10 minutes. An interindividual variation of the duration of the hyperemic plateau could influence the quantification of hyperemic MBF using ^15^O-water and could possibly explain some of the differences we found between regadenoson and adenosine hyperemic MBF. In a canine preclinical study, increasing the regadenoson injection duration to 30 seconds significantly prolonged the hyperemic plateau^31^ suggesting that a prolonged bolus could be more suitable than a rapid injection of regadenoson for methods that need several minutes for the quantification process.

In our study, we chose the timing carefully, based on the earlier study by Lieu et al.^14^ and our own simulations. One of our concerns was that the duration of the hyperemic plateau would not be sufficient for MBF quantification with ^15^O-water PET. However, our simulations showed that a hyperemic plateau duration of more than about 1 minute is sufficient for accurate estimation of MBF. Another concern with the use of regadenoson is the required time delay between regadenoson injection and tracer administration. With our choice of timing between regadenoson and radiotracer injections, which also was in line with the recommendations of the manufacturer, regadenoson was not inferior to adenosine in hyperemic response, in contrast to the findings of the earlier mentioned ^82^Rb PET study by Johnson and Gould^8^. Our simulations confirmed this, showing that a delay of 30 seconds is adequate.

There are several possible sources of variability of hyperemia between the adenosine and regadenoson PET in our study: data analysis, measurement technique, physiologic variation of MBF, or variability due to stressor. Repeatability of data analysis in aQuant is excellent as the analysis is nearly completely automated, with the only option for user intervention being the adjustment of the segmental VOIs. A newly accepted publication (Nordström et al, J Nucl Cardiol 2020) on the effects of PET-CT misalignment on MBF includes intra- and inter-observer variability of MBF analysis with aQuant which was of the order of a few percent only (mean inter-observer difference 0.7 ± 1.1% and mean intra-observer difference 0.5 ± 1.5% for whole myocardium, with the largest mean inter-observer differences seen in RCA at 1.6 ± 3.4%) and hence much smaller than the variability found in the present work. The variability of hyperemia between the adenosine and regadenoson PET in our study can at least to some degree be attributed to measurement technique and physiologic variations of myocardial blood flow. However, the variability in our study appears larger than the variability of repeated measurements of ^15^O-water myocardial perfusion at rest and during adenosine hyperemia as reported by Kaufman et al^32^. The repeatability coefficient for global adenosine stress MBF they reported was 0.90 mL·g·min. We found similar results in terms of reproducibility in a recent comparison between MBF measurements with ^15^O-water using PET-CT and PET-MR in the same patients 33, with reproducibility coefficients (RDC) of 0.84 and 0.92 mL·g·min at the global and regional level, respectively. The reproducibility coefficients (RDC) for MBF in the present study, as shown in Table 3, appear somewhat higher than in those previous two, which then could be attributed to differences in the responses to the stressors; but as the 95% confidence limits of the RDC are wide, this would need a larger number of patients to prove. In our study, we used three different and relatively old PET-CT systems. Newer PET-CT scanners with higher sensitivity and improved resolution could potentially give less variation in the MBF measurements and thus slightly smaller variability of hyperemia between adenosine and regadenoson PET.

**Table 3:** Reproducibility coefficients REGWATER

	MBF AD stress	MBF REG stress	Reproducibility coefficient (RDC) Absolute (CI) %	
Global	2.68 ± 0.80	2.76 ± 0.79	0.99 (0.77-1.40)	36%
Regional	2.64±0.92	2.66±0.94	1.17 (0.91-1.66)	45%

Reproducibility coefficient = 1.96 × SD of differences; absolute (with 95% confidence intervals) and % of mean MBF for adenosine and regadenoson.

Normal ranges and optimal cut-off limits have recently been defined for ^15^O-water PET and adenosine in diagnostic work in patients with suspected CAD.^13^ When quantification is applied in clinical work, it is critical to know whether the same cut-off values could be used also with regadenoson as with adenosine. Our study was relatively small; only 11 of the patients underwent invasive coronary angiography and the coronary lesions were assessed visually with only very few FFR measurements. Six patients were assessed with CTA only. Hence, no firm conclusions can be drawn on cut-off values for regadenoson in comparison with adenosine from our study. However, when using the previously defined cut-off value of 2.3 mL·min·g for stress MBF, the agreement between adenosine and regadenoson PET was substantial (Kappa coefficient = 0.67) on a patient level. On a regional level, the agreement between stressors was moderate (Kappa coefficient = 0.57), which is in line with the overall moderate agreement for the quantitative values for stress MBF. In most regions with discordant findings, the MBF value was close to the cut-off 2.30, as can be seen in Figure 4.

### Study Limitations

The subjects were asked to refrain from consumption of caffeine-containing substances for 24 hours before the PET examination, but serum concentrations of caffeine were not measured and it is possible that the vasodilating effect of adenosine and/or regadenoson was affected by caffeine intake prior to the perfusion studies. In a study by Banko et al., 19% of patients who negated recent caffeine ingestion still had detectable serum caffeine levels.^34^ While many previous studies indicate a significant influence of caffeine intake on cardiac perfusion measurements during adenosine- and dipyridamole-induced hyperemia,^35^ the effects of caffeine intake on regadenoson perfusion imaging remain unclear with contradictory results.^27^,^28^,^36^,^37^ In a canine study, caffeine dose dependently reduced the duration, but not the peak increase of coronary blood flow caused by regadenoson,^38^ which may affect blood flow results differently depending on modality and imaging or quantification method used.

In our study, two of the patients showed no hyperemic response to one of the vasodilators. It is possible that other patients with discordant findings between adenosine and regadenoson, but with less obvious discrepancies in hyperemic flow, also had remaining caffeine effects.

The adenosine and the regadenoson studies were not blinded to patients or staff, nor was the order randomized. However, no systematic differences were found between the adenosine and the regadenoson MBF results. Moreover, this was a relatively small study and it was performed in two centers, with two different PET scanners and some logistic/methodological differences between sites. Data from the two different sites were compared but there was no significant difference in MBF results between sites neither between stress scans or rest scans either for adenosine or regadenoson.

## New Knowledge Gained

The correlation between regadenoson- and adenosine-induced hyperemic MBF measured with ^15^O-water PET is good but the agreement is only moderate.

## Conclusion

Mean hyperemic MBF values did not differ significantly between adenosine and regadenoson. However, intra-individual differences between adenosine- and regadenoson-induced hyperemic MBF appear larger than those observed in test–retest studies with adenosine. Previously established cut-off values for ^15^O-water PET should be used cautiously if using regadenoson as vasodilator for assessment of myocardial perfusion in the detection of significant coronary artery disease.

## Supplementary Information

Below is the link to the electronic supplementary material.

Supplementary file1 (M4A 10228 kb)

Supplementary file2 (PPTX 7160 kb)

## Abbreviations

CAD: Coronary artery disease
CTA: Computed tomography angiography
ICA: Invasive coronary angiography
MBF: Myocardial blood flow
MFR: Myocardial flow reserve
MRI: Magnetic resonance imaging
PET: Positron emission tomography
PTF: Perfusable tissue fraction
SPECT: Single Photon Computed Tomography
