# Supplementary material for: Quantitative myocardial perfusion response to adenosine and regadenoson in patients with suspected coronary artery disease
Source: J Nucl Cardiol. 2021 Aug 12;29(1):24–36. doi: 10.1007/s12350-021-02731-6 (PMC8873130; doi:10.1007/s12350-021-02731-6)
Supplement: Supplementary file 2 — Supplementary file2 (PPTX 7160 kb) [file 12350_2021_2731_MOESM2_ESM.pptx]

## Slide 1
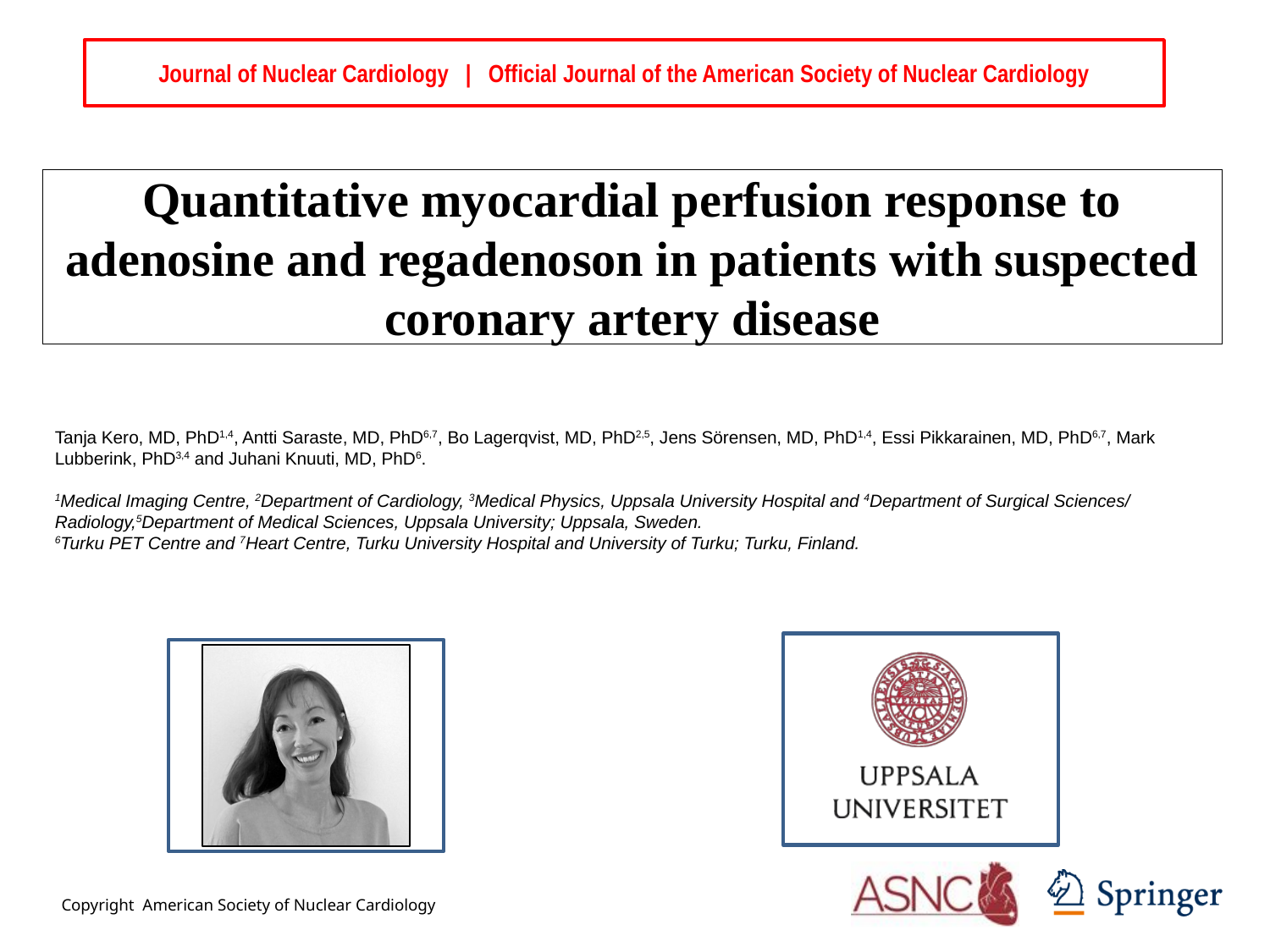

Journal of Nuclear Cardiology | Official Journal of the American Society of Nuclear Cardiology
# Quantitative myocardial perfusion response to adenosine and regadenoson in patients with suspected coronary artery disease
Tanja Kero, MD, PhD1,4, Antti Saraste, MD, PhD6,7, Bo Lagerqvist, MD, PhD2,5, Jens Sörensen, MD, PhD1,4, Essi Pikkarainen, MD, PhD6,7, Mark Lubberink, PhD3,4 and Juhani Knuuti, MD, PhD6.
1Medical Imaging Centre, 2Department of Cardiology, 3Medical Physics, Uppsala University Hospital and 4Department of Surgical Sciences/ Radiology,5Department of Medical Sciences, Uppsala University; Uppsala, Sweden.
6Turku PET Centre and 7Heart Centre, Turku University Hospital and University of Turku; Turku, Finland.
Head shot of author
required
Copyright American Society of Nuclear Cardiology

## Slide 2
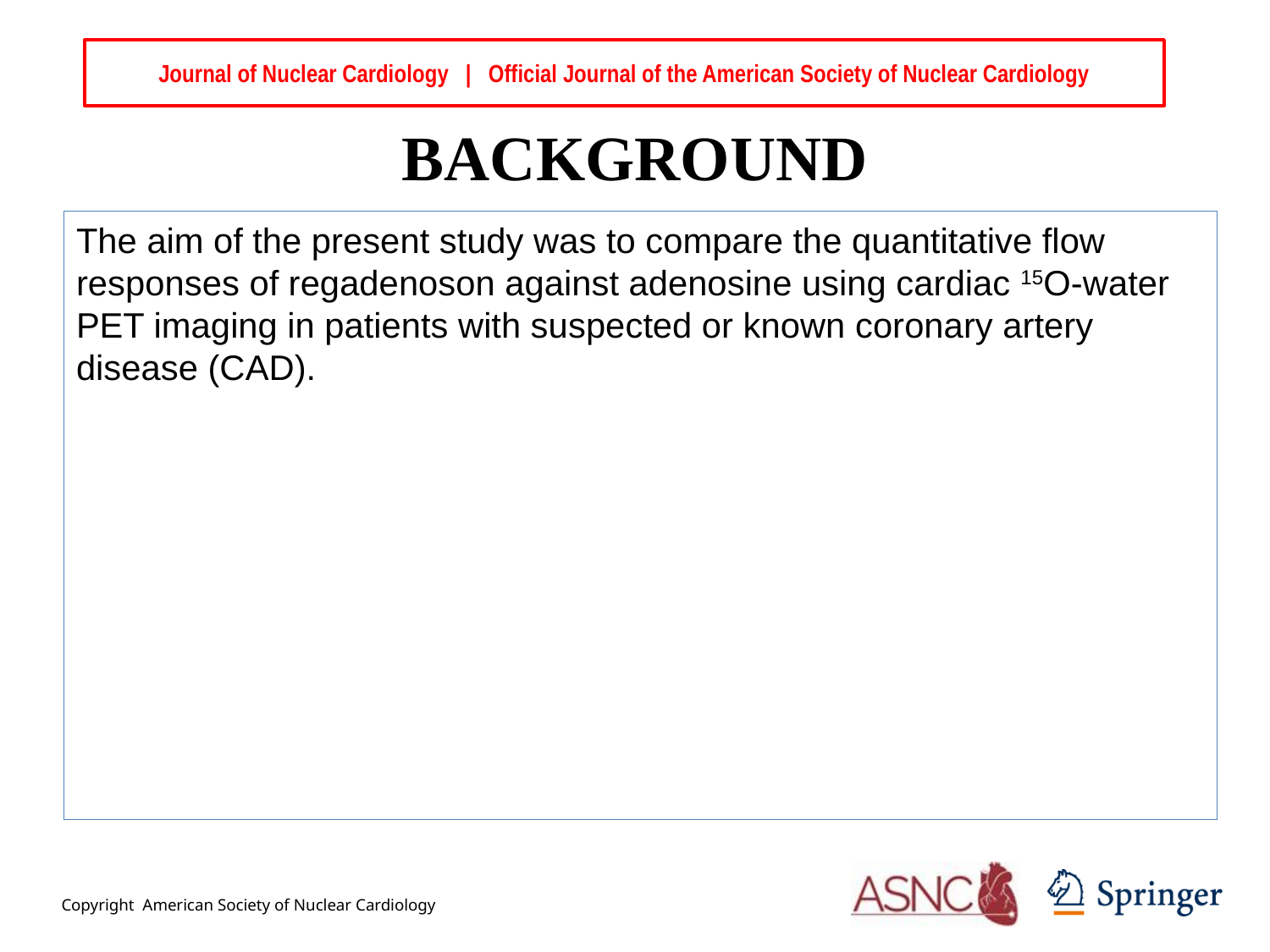

Journal of Nuclear Cardiology | Official Journal of the American Society of Nuclear Cardiology
# BACKGROUND
The aim of the present study was to compare the quantitative flow responses of regadenoson against adenosine using cardiac 15O-water PET imaging in patients with suspected or known coronary artery disease (CAD).
Copyright American Society of Nuclear Cardiology

## Slide 3
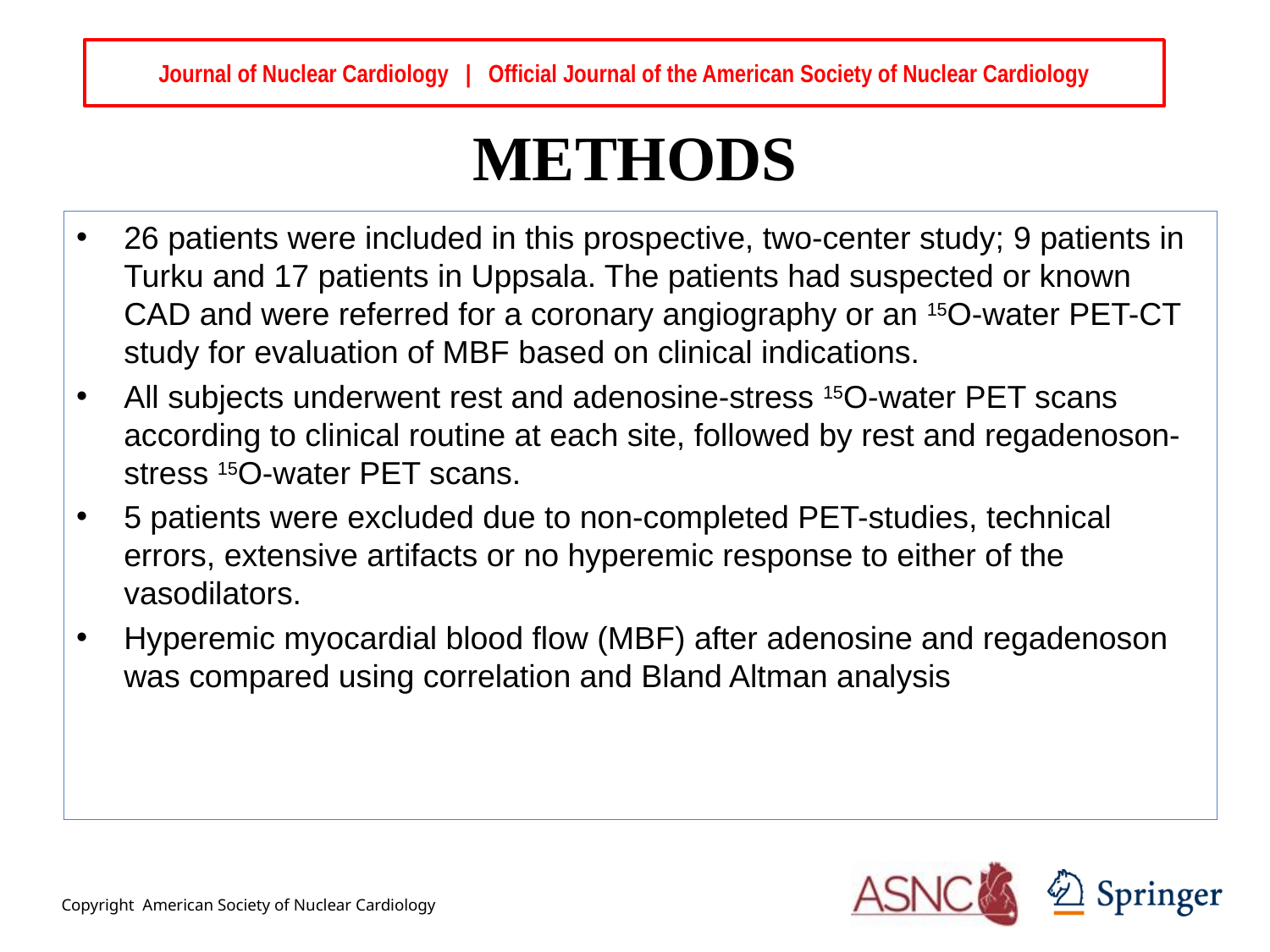

Journal of Nuclear Cardiology | Official Journal of the American Society of Nuclear Cardiology
# METHODS
26 patients were included in this prospective, two-center study; 9 patients in Turku and 17 patients in Uppsala. The patients had suspected or known CAD and were referred for a coronary angiography or an 15O-water PET-CT study for evaluation of MBF based on clinical indications.
All subjects underwent rest and adenosine-stress 15O-water PET scans according to clinical routine at each site, followed by rest and regadenoson-stress 15O-water PET scans.
5 patients were excluded due to non-completed PET-studies, technical errors, extensive artifacts or no hyperemic response to either of the vasodilators.
Hyperemic myocardial blood flow (MBF) after adenosine and regadenoson was compared using correlation and Bland Altman analysis
Copyright American Society of Nuclear Cardiology

## Slide 4
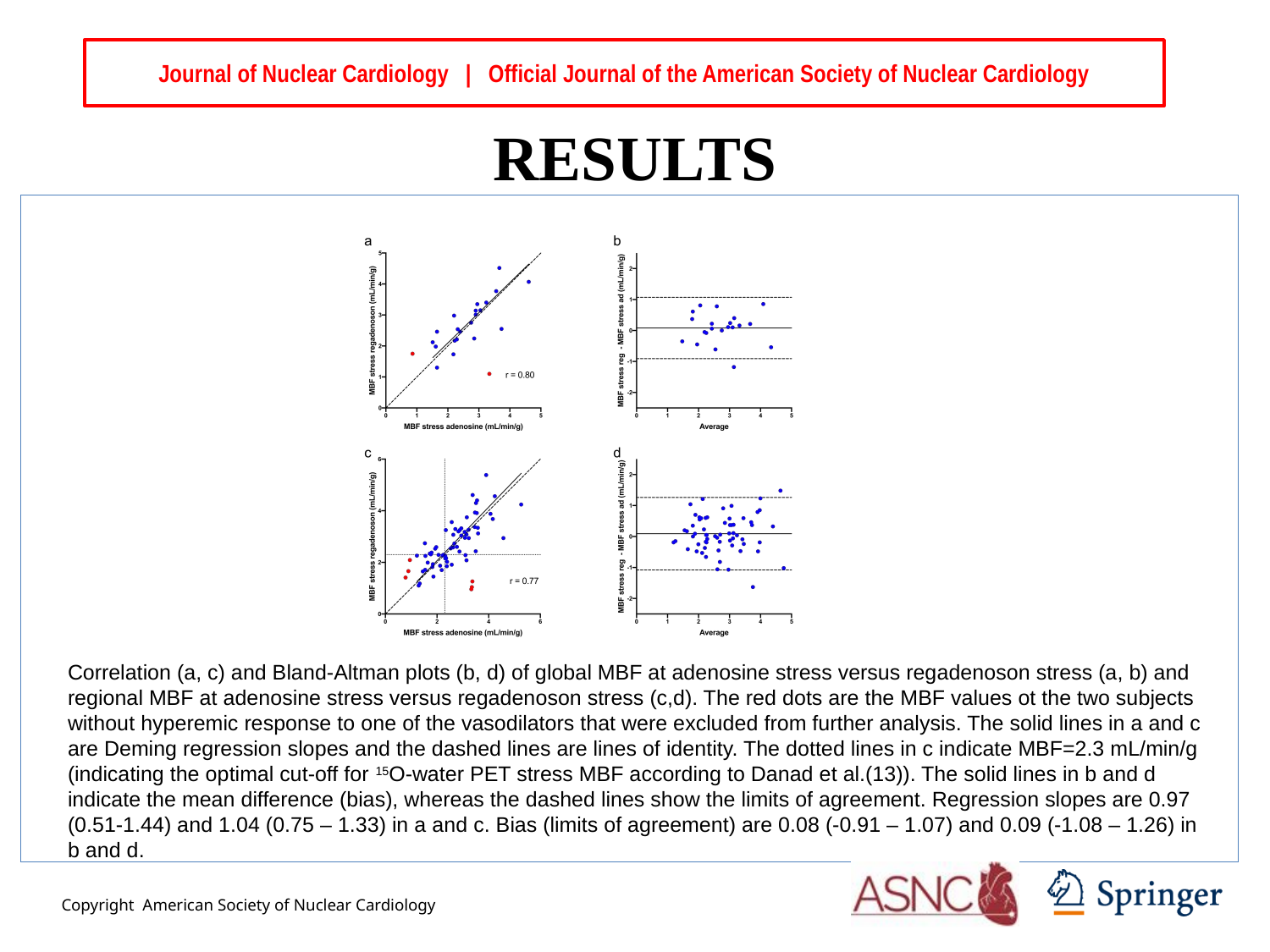

Journal of Nuclear Cardiology | Official Journal of the American Society of Nuclear Cardiology
# RESULTS
Correlation (a, c) and Bland-Altman plots (b, d) of global MBF at adenosine stress versus regadenoson stress (a, b) and regional MBF at adenosine stress versus regadenoson stress (c,d). The red dots are the MBF values ot the two subjects without hyperemic response to one of the vasodilators that were excluded from further analysis. The solid lines in a and c are Deming regression slopes and the dashed lines are lines of identity. The dotted lines in c indicate MBF=2.3 mL/min/g (indicating the optimal cut-off for 15O-water PET stress MBF according to Danad et al.(13)). The solid lines in b and d indicate the mean difference (bias), whereas the dashed lines show the limits of agreement. Regression slopes are 0.97 (0.51-1.44) and 1.04 (0.75 – 1.33) in a and c. Bias (limits of agreement) are 0.08 (-0.91 – 1.07) and 0.09 (-1.08 – 1.26) in b and d.
Copyright American Society of Nuclear Cardiology

## Slide 5
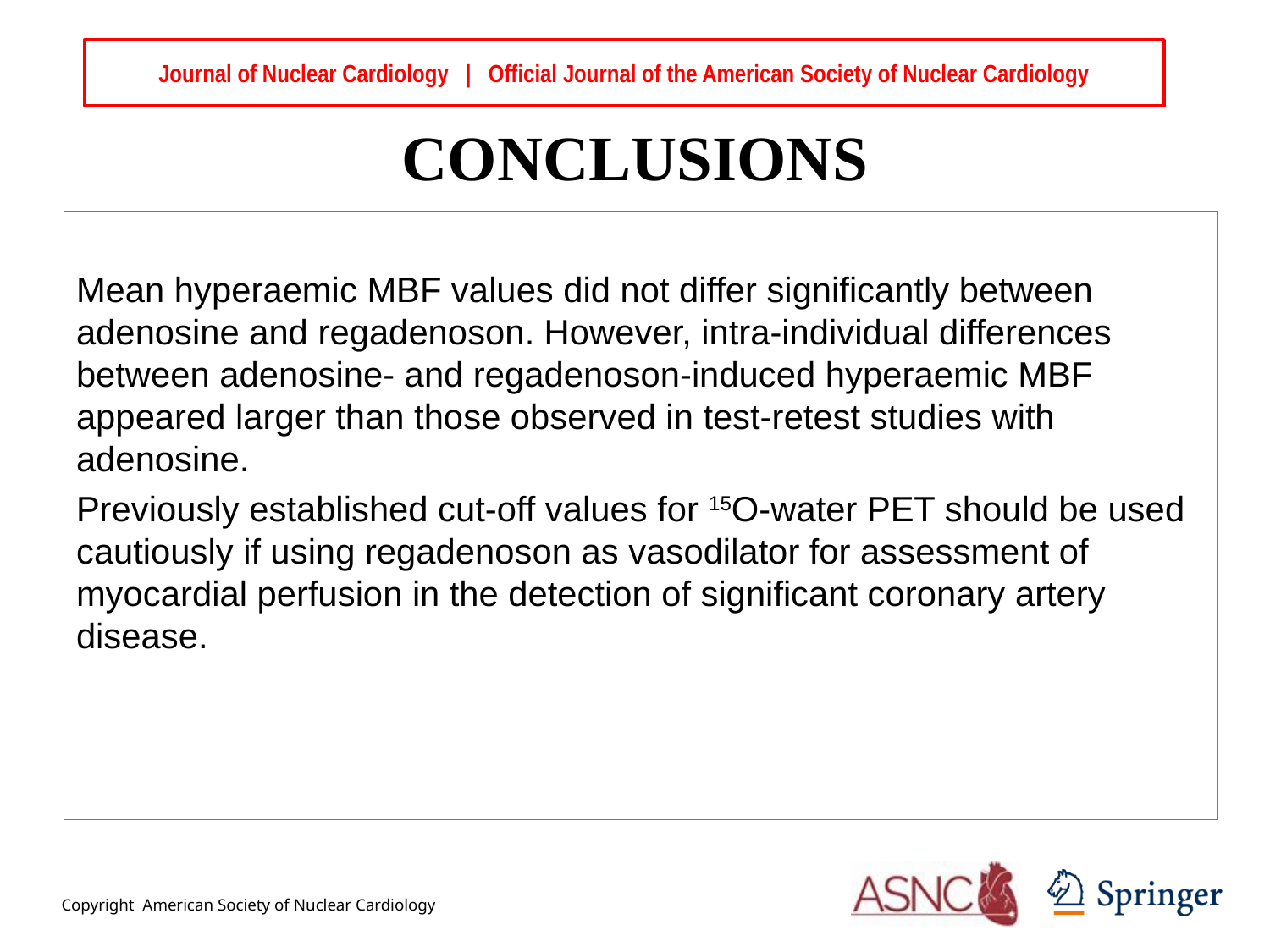

Journal of Nuclear Cardiology | Official Journal of the American Society of Nuclear Cardiology
# CONCLUSIONS
Mean hyperaemic MBF values did not differ significantly between adenosine and regadenoson. However, intra-individual differences between adenosine- and regadenoson-induced hyperaemic MBF appeared larger than those observed in test-retest studies with adenosine.
Previously established cut-off values for 15O-water PET should be used cautiously if using regadenoson as vasodilator for assessment of myocardial perfusion in the detection of significant coronary artery disease.
Copyright American Society of Nuclear Cardiology
